# The Serum Metabolic Biomarkers in Early Diagnosis and Risk Stratification of Acute Coronary Syndrome

**DOI:** 10.3389/fphys.2020.00776

**Published:** 2020-07-21

**Authors:** Huali Jiang, Hualong Jiang, Jian Zhang, Weijie Chen, Changyou Luo, Heng Li, William Hau, Benfa Chen, Shanhua Wang

**Affiliations:** ^1^Department of Cardiovascular, Tungwah Hospital of Sun Yat-sen University, Dongguan, China; ^2^Department of Urology, Tungwah Hospital of Sun Yat-sen University, Dongguan, China; ^3^Department of Radiation Oncology, Affiliated Cancer Hospital & Institute of Guangzhou Medical University, Guangzhou, China; ^4^State Key Laboratory of Respiratory Diseases, Guangzhou Institute of Respiratory Disease, Affiliated Cancer Hospital & Institute of Guangzhou Medical University, Guangzhou, China; ^5^Department of Medicine and Therapeutics, Faculty of Medicine, The Chinese University of Hong Kong, Shatin, Hong Kong

**Keywords:** serum, metabolomics, biomarkers, acute coronary syndrome, liquid chromatography, mass spectrometry

## Abstract

Despite advances in the treatment of coronary diseases, acute coronary syndrome (ACS) remains the leading cause of death worldwide. ACS is associated with metabolic abnormalities of lipid oxidation stress. In this study, based on liquid chromatograph mass spectrometry technique, we conducted the metabolic profiling analysis of serum samples from stable plaques (SPs) and vulnerable plaques (VPs) in ACS patients for exploring the potential biomarkers of plaque stability. The results showed that four differential metabolites were identified between the SPs and VPs, including betaine, acetylcarnitine, 1-heptadecanoyl-sn-glycero-3-phosphocholine, and isoundecylic acid. Meanwhile, the diagnostic model was identified using stepwise logistic regression and internally validated with 10-fold cross-validation. We analyzed the correlations between serum metabolic perturbations and plaque stability, and the serum betaine and ejection fraction-based model was established with a good diagnostic efficacy [area under the curve (AUC) = 0.808, sensitivity = 70.6%, and specificity = 80.0%]. In summary, we firstly illustrate the comprehensive serum metabolic profiles in ACS patients, suggesting that the combined model of serum betaine and ejection fraction seems to be used as the potential diagnostic biomarker for the vulnerability of plaque stability.

## Introduction

Coronary artery disease (CAD), one of the metabolic disorder diseases, causes the most serious cardiovascular death events in the world (Terashima et al., 2000). Acute coronary syndrome (ACS), a severe category of CAD, is usually associated with the rupture of unstable plaques (VPs), formation of thrombosis, and occurrence of acute myocardial ischemia (Goodacre et al., 2009). Clinically, ACS can be distinguished into unstable angina (UA) and acute myocardial infarction (AMI). Early detection of VPs is critical to receive suitable timely therapy and inhibit the happening of AMI and heart failure (Reihani et al., 2018).

Previous studies suggested that atherosclerosis is a chronic inflammatory arterial disease and led by an imbalanced lipid metabolism, and subsequent impaired immune response in developing over decades, though a number of biomarkers related to the peripheral or coronary diseases have been found to be associated with lipid metabolism and might be useful in predicating ACS (Ridker, 2007; Wang et al., 2007; Antoniades et al., 2009). However, most of the studies on biomarkers of coronary diseases were based on retrospective study, and the predictive sensitivity and specificity of biomarkers remain to be nonconforming and inconclusive. Thus, exploration of novel circulatory biomarkers capable of predicting cardiovascular events induced by VPs is still a major challenge.

Metabolomics, a newly developed discipline, is considered to allow qualitative and quantitation analysis of low-molecular-weight metabolites or endogenous metabolic substances in physiological or pathological conditions (Calderon-Santiago et al., 2017). Metabolomics is widely used in diagnosing a variety of metabolic diseases, such as gastritis (Chen et al., 2019), liver fibrosis (Fang et al., 2017), diabetes (Klein and Shearer, 2016), and cancers (Huang et al., 2013; Chan et al., 2014; O’Keefe et al., 2015). Simultaneously, the application of metabolomics in cardiovascular diseases like hyperlipidemia (Sun et al., 2017), atherosclerosis (Wishart, 2016), and hypertension (Au et al., 2017) has also gradually attracted much more attention. However, the metabolomics change of ACS is not well known and the risk stratification role of serum metabolic biomarkers on ACS and stable CAD need to be further explored.

In the present study, we explored the metabolomics on serum samples of stable plaques (SPs) and VPs in ACS patients to analyze the metabolomics correlations between SPs and VPs and to find and identify specific metabolites that may be potentially conducted as risk stratification biomarkers of ACS patients.

## Materials and Methods

### Patients’ Characteristics and Sample Selection

In this study, we reviewed and identified patients with newly diagnosed ACS who were treated between November 2016 and February 2019 at the Tungwah Hospital of Sun Yat-sen University. We recruited 252 adult patients who present with acute chest pain and age >20 years old in the emergency department of the Tungwah Hospital of Sun Yat-sen University. Exclusion criteria are as follows: patients with severe liver or kidney diseases, marrow and hematological system diseases, chest pain caused by trauma, malignancy, or previously diagnosed with coronary disease in 2 months and/or were treated accordingly. All participants with ACS were enrolled based upon typical clinical features, electrocardiogram (ECG) examination, elevated cardiac troponin I (cTnI), coronary angiography (CAG), and intravenous ultrasound (IVUS) that met the criteria of ACS from guideline recommendations. This study was approved by the ethics committee of Tungwah Hospital of Sun Yat-sen University. Informed consent was obtained from all subjects before enrollment in the study. Fresh serum samples (around 800 μl) were collected after enrollment, according to the manufacturer’s protocol of Longseegen storage kit (Longsee Biomedical Corporation, Guangzhou, China), and stored at −80°C.

### Chemicals

Liquid chromatograph mass spectrometer (LC-MS) grade acetonitrile, methanol, and ultrapure water were purchased from Thermo Fisher Scientific (Waltham, MA, United States). Ammonium acetate and formic acid were purchased from CNW (Shanghai, China). 1-Heptadecanoyl-sn-glycero-3-phosphocholine and betaine were from ChromaBio (Chengdu, China). Acetylcarnitine was purchased from EFFBIO (Shanghai, China).

### Sample Preparation

Blood samples from ACS patients were collected and homogenized. Serum sample was collected, which was added with 800 μl methanol/acetonitrile. Then, the sample was vibrated, subjected to ultrasound, and incubated at −20°C to promote protein mixture precipitation. The serum mixtures were centrifuged, and the supernatants were collected, vacuum-dried, and re-dissolved. Lastly, the supernatants were conducted to metabolomics profiling by liquid chromatography mass spectrometry.

### Untargeted Metabolomics and Measurement of Metabolites

Ninety-eight serum samples from ACS patients were assigned to detect untargeted metabolomics. After the samples were screened by propensity score matching, 65 serum samples (SPs, *n* = 33; VPs, *n* = 32) were included to perform untargeted metabolomics. Followed by previous protocols with modifications (Chen et al., 2016, 2017; Shivanna et al., 2016; Zhang et al., 2019), after serum sample preparation, metabolite preprocessing and statistical analysis were performed with a Waters Acquity^TM^ Ultra Performance.

The process was as follows: 95% A (a acetonitrile solution) and 5% B (a water solution) from 0 to 0.5 min, 90% A and 10% B from 0.5 to 2 min, 40% A and 60% B from 2 to 10 min, 5% A and 95% B from 10 to 14 min, 5% A and 95% B from 14 to 16 min, 95% A and 5% B from 16 to 18 min and 95% A and 5% B from 18 to 20 min.

### Targeted Metabolomics and Measurement of Serum Biomarkers

One hundred fifty-four serum samples from ACS patients were assigned to detect targeted metabolomics. After the samples were screened by propensity score matching, 101 serum samples (SPs, *n* = 51; VPs, *n* = 50) were included to perform targeted metabolomics. The process was as follows: flow rate was kept at 0.5 ml/min with a 3-min run by isocratic elution. All samples were analyzed in positive mode. The parameters of ion spray voltage, source temperature, and the MS1 scan range and the MS2 are the same as untargeted metabolomics and the measurement of metabolites.

### Metabolites Identification

As the protocol was described by the reported procedure (Zhao et al., 2012, 2013), the metabolite was analyzed. Based on the molecular mass data of metabolite, all putative identities were confirmed by matching with entries in the METLIN^1^, the HMDB database^2^, and KEGG database^3^. The metabolite would be identified on the condition that the mass difference between the observed value and the database value was <0.025 Da.

### Metabolomics Data Analysis

Untargeted metabolomics datasets were analyzed by R with packages MetaboAnalyst. To reduce the metabolite concentration differences between samples and make the skewed distributions more symmetric, the data were normalized and non-linear conversed by log transformations.

### Statistical Analysis

For univariate analysis, the statistical significance of features was analyzed between the VPs group and the SPs group using *t*-test. For multivariate analysis, partial-least-squares discrimination analysis (PLS-DA) was applied to reduce the effect of inter-subject variability and identify differential metabolites that significantly contributed to plaque stability. To ensure the quality of the multivariate model and avoid the risk of over-fitting, according to the variable importance in the projection (VIP) scores, the metabolites were further ranked based on the PLS-DA model. Metabolites with VIP scores >1.0 are considered as the significant contributors. *P < 0.05 and **P < 0.01 were considered to be statistically significant.

Stepwise logistic regression on dichotomized (positive/negative) data in SAS was used to narrow the number of metabolites. Based on the results of this logistic regression, the diagnostic accuracy was further performed based on the area under the curve (AUC). Models were internally validated using 10-fold cross-validation, meaning that the data were split into 10 equally sized datasets. The AUC was then calculated using each of the 10 datasets for validation in turn and the remaining 9 datasets for training. The average of the 10 results (i.e., average of the AUC calculated from the 10 validation subsets) is reported as a value for the 10-fold cross-validation.

## Results

### Demographic Characteristics of the Study Population

**FIGURE 1:**
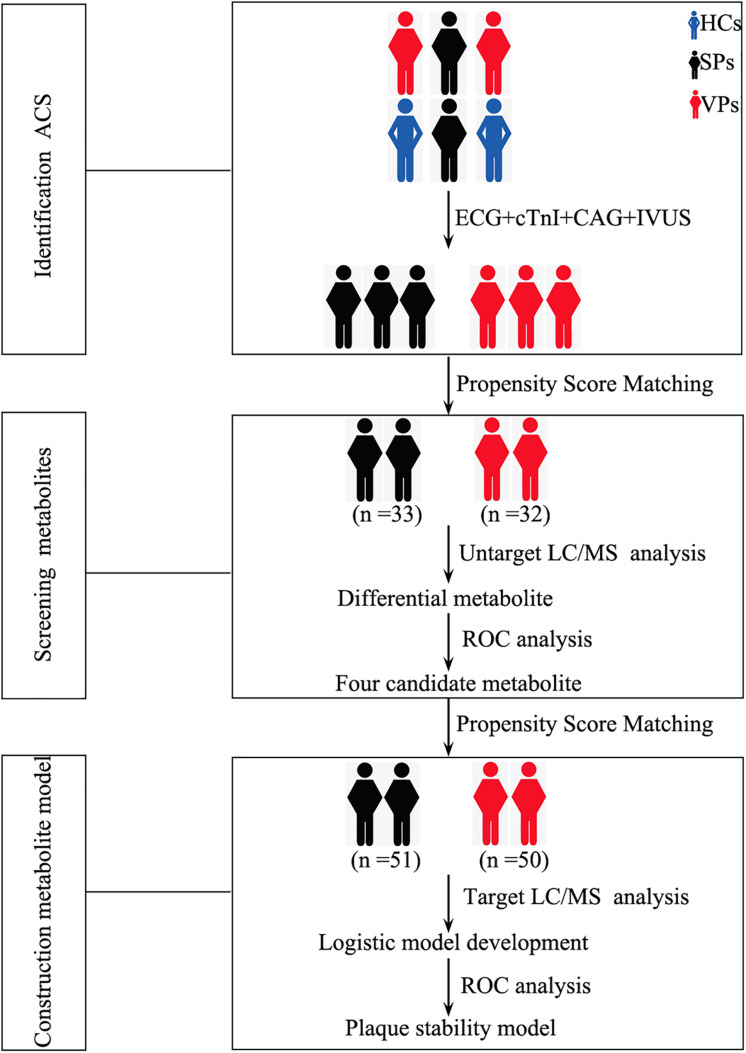
Study design for the development of metabolite-based model.

The populations are diagnosed as ACS by ECG, cTnI, and CAG. The stability of atherosclerotic plaques is detected by intravenous ultrasound (IVUS). The general clinical and demographic data on ACS patients are presented in Table 1. Atherosclerotic plaque and lesion were significantly associated with ACS. There were no significant differences in age, gender, height, weight, smoking, drinking, hypertension, diabetes, and blood biochemistry between the VPs group and SPs group by Student’s *t*-test. The flow diagram of the overview of study design was depicted in Figure 1.

**TABLE 1:** Characteristics of the population in untargeted metabolomics analysis groups.

**Characteristics**	**SPs (*n* = 33)**	**VPs (*n* = 32)**	***P***
Age, y	56.58 (10.52)	60.19 (10.49)	0.252
Male Sex, *n* (%)	22 (66.7)	23 (71.9)	0.629
Height, cm	162.88 (8.24)	161.69 (7.60)	0.832
Weight, kg	68.33 (12.27)	62.97 (9.45)	0.165
Smoking, *n* (%)	9(27.3)	13 (40.6)	0.512
Drinking, *n* (%)	2 (6.1)	4 (12.5)	0.668
Hypertension, *n* (%)	20 (60.6)	24 (75.0)	0.463
Diabetes, *n* (%)	8 (24.2)	9 (28.1)	0.918
CREA μmol/L	77.48 (17.61)	92.06 (32.70)	0.064
URIC μmol/L	385.27 (90.27)	405.91 (135.69)	0.48
TCHO mmol/L	4.40 (0.90)	4.40 (1.10)	0.734
TG mmol/L	1.80 (1.28)	1.81 (1.15)	0.5
HDLC mmol/L	1.14 (0.36)	1.11 (0.24)	0.773
LDLC mmol/L	3.00 (1.52)	3.06 (0.86)	0.734
EF (%)	63.27 (7.87)	64.66 (6.52)	0.42
Plaque (%)			**> 0.001**
0	33 (100.0)	0 (0.0)	
1	0 (0.0)	32 (100.0)	
NA	0 (0.0)	0 (0.0)	
Lesion (%)			**> 0.001**
0	2 (6.1)	6 (18.8)	
1	23 (69.7)	18 (56.2)	
2	4 (12.1)	5 (15.6)	
3	4 (12.1)	3 (9.4)	
NA	0 (0.0)	0 (0.0)	

*SP, stable plaque; VP, vulnerable plaque; HC, healthy control; CREA, creatinine; URIC, uric acid; TCHO, total cholesterol; TG, triglyceride; HDLC, high-density lipoprotein cholesterol; LDLC; low-density lipoprotein cholesterol; EF, ejection fraction. Continuous variables are presented as mean (SD). Categorical variables are presented as *n* (%). The bold values of Plaque (%) and lesion (%) mean the plaque and lesion are only two different factors in SP and VP groups.*

### Metabolomics Analysis of Serum Samples by Untargeted LC/MS

**FIGURE 2:**
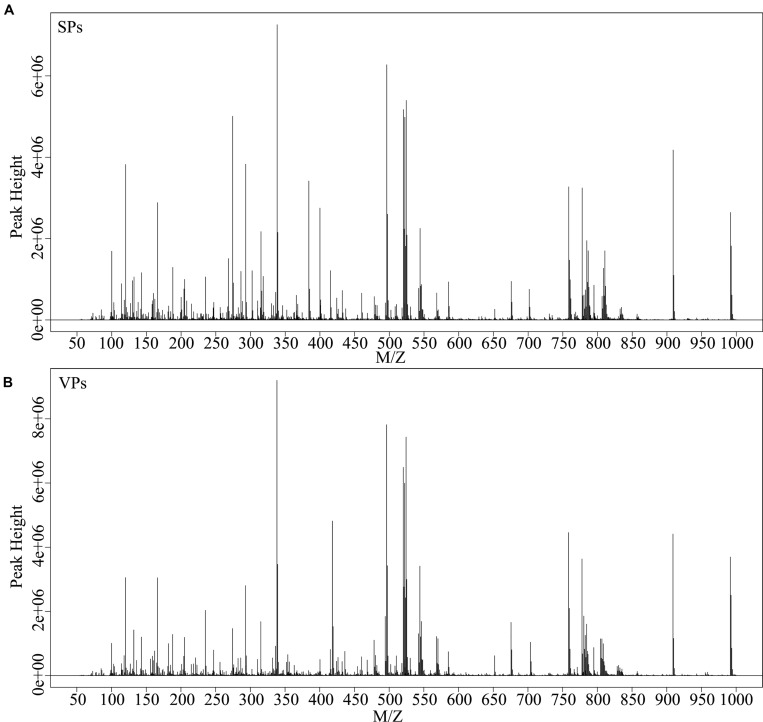
Typical mass spectra of the SPs group **(A)** and VPs group **(B)**. SPs, stable plaque groups; VPs, vulnerable plaque groups.

**FIGURE 3:**
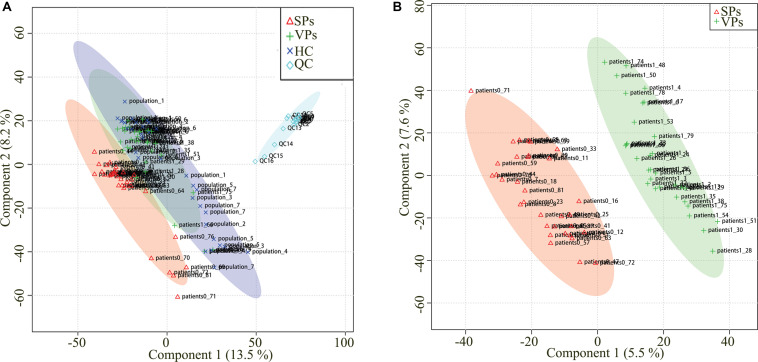
Partial least squares discriminant analysis (PLS-DA) of serum metabolomics data. Serum metabolomics data for quality control **(A)**. Serum metabolites distinguished SPs group and VPs group **(B)**. ACS, acute coronary syndrome; HC, healthy controls; VPs, vulnerable plaque groups; SPs, stable plaque groups; QC, quality control.

Representative mass spectrum images of SPs and VPs patients were shown in Figures 2A,B. Using untargeted LC/MS, we detected the variables positive ion mode and negative ion mode. Three thousand sixty-nine molecular metabolites were obtained and then subjected to statistical analysis using MetaboAnalyst (Supplementary Table S1). PLS-DA analysis found that HCs and ACS patients, especially in SPs and VPs patients, showed differential distributions with *Q*^2^ of 0.820 and *R*^2^ of 0.973, which means that the model was not only overlifting but also reliable (Figures 3A,B).

**FIGURE 4:**
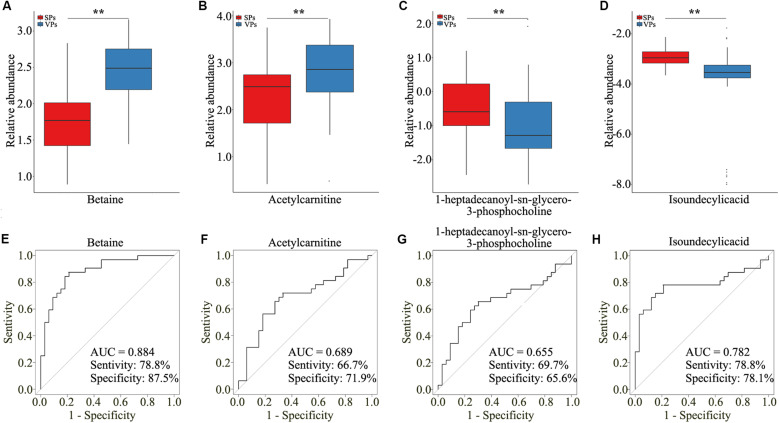
The four selected metabolites detected by untargeted LC/MS analysis. **(A–D)** Boxplots showed the relative abundance of the four metabolites in serum including betaine **(A)**, acetylcarnitine **(B)**, 1-heptadecanoyl-sn-glycero-3-phosphocholine **(C)**, and isoundecylic acid **(D)**. **(E–H)** Analysis of PLS-DA-based ROC curves of betaine **(E)**, acetylcarnitine **(F)**, 1-heptadecanoyl-sn-glycero-3-phosphocholine **(G)**, and isoundecylic acid **(H)** in VPs and SPs groups. VP, vulnerable plaque groups; SP, stable plaque groups.

To explore the clinical metabolites, we screened the differential metabolites in VPs and SPs by receiver operating characteristic curve (ROC) >0.6. The four metabolites are presented in Table 2. Four metabolites including betaine, acetylcarnitine, 1-heptadecanoyl-sn-glycero-3-phosphocholine, and isoundecylic acid were significantly associated with the clinical plaque stability of ACS patients (Figures 4A–D). The curve values (AUC) of the four metabolites in VPs vs. SPs groups were 0.884, 0.689, 0.655, and 0.782, the sensitivity were 78.8, 66.7, 69.7, and 78.8%, and the specificity were 87.5, 71.9, 65.6, and 78.1% (Figures 4E–H). These results indicated that the four metabolites played important roles in the stability of plaques in ACS patients.

**TABLE 2:** List of Metabolite identified in combining untargeted and targeted metabolomics approaches.

**Metabolite**	**Rt (min)**	***M/Z***	**Formula**	**HMDB ID**
Betaine	0.87	118.09	C_5_H_11_NO_2_	HMDB0000043
Acetylcarnitine	0.97	204.12	C_9_H_7_NO	HMDB0000201
1-heptadecanoyl-sn-glycero-3-phosphocholine	12.91	510.36	C_16_H_30_N_6_O_4_S_1_	HMDB0012108
Isoundecylic acid	12.07	213.19	C_13_H_26_O_2_	–

### Metabolomics Analysis of Serum Samples by Targeted LC/MS

**FIGURE 5:**
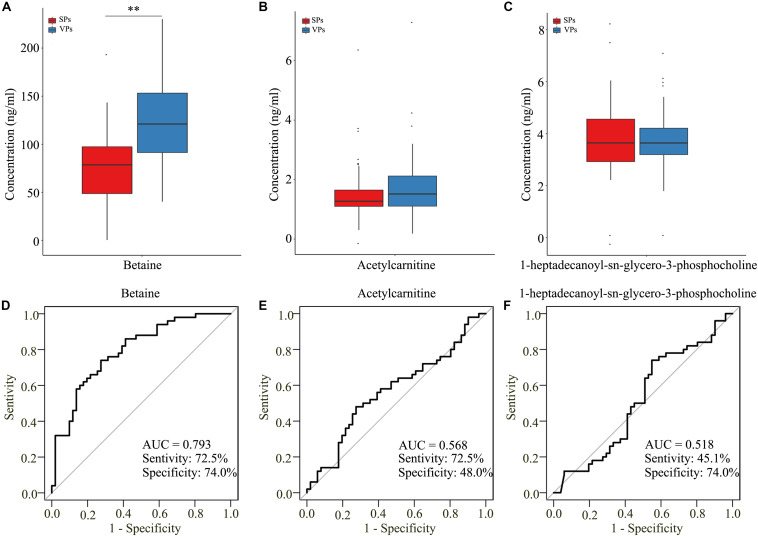
The three metabolites detected by target LC/MS analysis. **(A–C)** Boxplots showed the concentration of betaine **(A)**, acetylcarnitine **(B)**, and 1-heptadecanoyl-sn-glycero-3-phosphocholine **(C)**. **(D–F)** Analysis of PLS-DA-based ROC curves of betaine **(D)**, acetylcarnitine **(E)**, and 1-heptadecanoyl-sn-glycero-3-phosphocholine **(F)** in VPs and SPs groups. VPs, vulnerable plaque groups; SPs, stable plaque groups.

To further detect the value of candidate metabolites, we detected them by targeted LC/MS analysis. As shown in Table 3, the clinical characteristics of VPs and SPs groups were matched. As isoundecylic acid was not found in the human metabolome database (HMDB), we systematically analyzed three metabolites’ (betaine, acetylcarnitine, 1-heptadecanoyl-sn-glycero-3-phosphocholine) relative concentration and found that betaine was significantly upregulated in VPs, which was consistent with the expression of betaine in untargeted LC/MS analysis (Figures 5A–C) and indicated that betaine was significantly related to the plaque stability of ACS patients. The AUC values of the three metabolites in VPs vs. SPs groups were 0.793, 0.568, 0.518, the sensitivity in two groups were 72.5%, 72.5%, 45.1% and the specificity were 74.0, 48.0, 65.6, and 74.0% (Figures 5D–F). These results indicated that the three metabolites, especially betaine, may predict the plaque stability.

**TABLE 3:** Characteristics of the population in targeted metabolomics analysis groups.

**Characteristics**	**SP (*n* = 51)**	**VP (*n* = 50)**	***P***
Age, y	57.10 (11.47)	59.46 (11.65)	0.446
Male Sex, *n* (%)	34 (66.7)	36 (72)	0.576
Height, cm	162.86 (8.11)	162.60 (7.71)	0.968
Weight, kg	67.53 (12.44)	65.28 (10.36)	0.582
Smoking, *n* (%)	17 (33.3)	19 (38.0)	0.867
Drinking, *n* (%)	3 (5.9)	7 (14.0)	0.394
Hypertension, *n* (%)	30 (58.8)	29 (58.0)	0.969
Diabetes, *n* (%)	12 (23.5)	15 (30.0)	0.831
CREA μmol/L	84.23 (29.22)	86.71 (30.58)	0.064
URIC μmol/L	388.39 (102.33)	390.32 (125.20)	0.852
TCHO mmol/L	4.42 (1.02)	4.33 (1.16)	0.773
TG mmol/L	1.98 (1.94)	1.74 (1.12)	0.269
HDLC mmol/L	1.22 (0.54)	1.11 (0.26)	0.366
LDLC mmol/L	2.94 (1.44)	3.03 (0.89)	0.615
EF (%)	63.10 (7.92)	65.38 (6.44)	0.295
Plaque (%)			**> 0.001**
0	51 (100.0)	0 (0.0)	
1	0 (0.0)	50 (100.0)	
NA	0 (0.0)	0 (0.0)	
Lesion (%)			**> 0.001**
0	3 (5.9)	8 (16.0)	
1	32 (69.7)	26 (52.0)	
2	9 (17.6)	10 (20.0)	
3	7 (13.7)	6 (12.0)	
NA	0 (0.0)	0 (0.0)	

*SP, Stable plaque; VP, vulnerable plaque; HC, healthy control; CREA, creatinine; URIC, uric acid; TCHO, total cholesterol; TG, triglyceride; HDLC, high-density lipoprotein cholesterol; LDLC, low-density lipoprotein cholesterol; EF, ejection fraction. Continuous variables are presented as mean (SD). Categorical variables are presented as *n* (%). The bold values of Plaque (%) and lesion (%) mean the plaque and lesion are only two different factors in SP and VP groups.*

### Development of a Metabolite-Based Model

**FIGURE 6:**
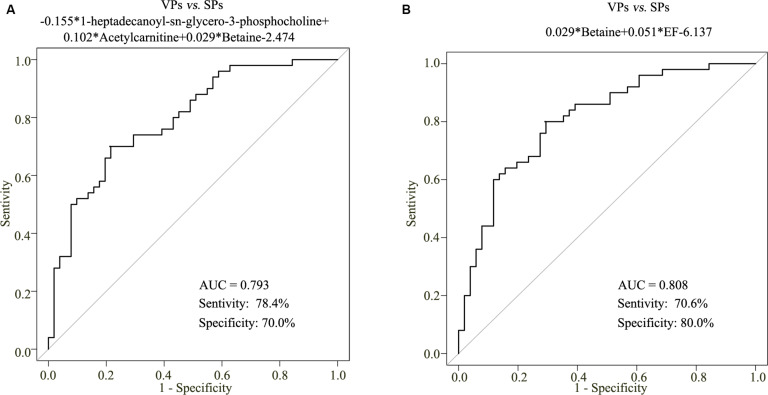
The metabolite-based model on plaque stability. ROC curve analysis of the three metabolite-based diagnostic models in VPs vs. SPs groups **(A)**. ROC curve analysis of the metabolite-based diagnostic model in distinguishing VPs and SPs groups from controls in the ACS patients **(B)**. VPs, vulnerable plaque groups; SPs, stable plaque groups.

To construct a diagnostic model that could be used to identify the stability of plaque with ACS patients, the sensitivity and specificity values of the betaine, acetylcarnitine, and 1-heptadecanoyl-sn-glycero-3-phosphocholine in the diagnosis of ACS and risk stratification of plaque stability were explored by logistic regression statistical analysis. However, the metabolite-based model in VPs vs. SPs groups was developed and the AUC values of VPs vs. SPs groups were 0.793, with the corresponding sensitivity of 78.4% and specificity of 70.0% (Figure 6A).

To improve the diagnostic performance of plaque stability, step-wise backward selection analysis was further performed to determine the best model in subgroups shown in Figure 6B. The model was presented as follows: logit (*P* = VPs vs. SPs) = 0.029*betaine + 0.051*EF-6.137 in VPs vs. SPs group. The AUC value was 0.808, with the corresponding sensitivity of 70.6% and specificity of 80.0%. These results demonstrated that the combined model was reliable and could be applied to differentiate the stability of plaque.

## Discussion

In this study, we systematically and comprehensively analyzed the serum metabolomics change on atherosclerotic plaque stability. The metabolite profiles of serum samples allowed classification of SPs and VPs patients. Four metabolites were significantly altered in ACS patients. Compared to SPs, betaine, acetylcarnitine, 1-heptadecanoyl-sn-glycero-3-phosphocholine, and isoundecylic acid were perturbed in VPs patients. Furthermore, the combined model of serum betaine and EF could be used as the potential diagnostic biomarker for distinguishing VPs patients from SPs.

Our study was firstly to identify the combination of betaine and clinical indicators that could be used for the detection of plaque stability. Betaine, a natural compound that exists in many organisms, can not only regulate cells against water osmotic or retention (Craig, 2004) but also increase energy metabolism by acting as a methyl donor (Alirezaei et al., 2012). Betaine also plays a potential antioxidant role in animals by increasing plasma glutathione peroxidase (Alirezaei et al., 2015). Oxidative stress is an important pathological process of atherosclerosis. Jiang et al. found that betaine played an important role on diabetic-induced oxidative stress-mediated p38 MAPK pathways (Jiang et al., 2019). Saeed et al. (2017) also identified that betaine could act as a natural anti-heat stress agent. Our results found that betaine was significantly upregulated in VPs patients and indicated that betaine played an important role on plaque stability, which was consistent with the protective role of betaine on organisms.

Acetylcarnitine, an index of energy substrate oxidation, could increase transportation of acetyl-CoA into the mitochondria during fatty acid oxidation. Pouralijan Amiri et al. found that acetylcarnitine was up-regulated in UA (Pouralijan Amiri et al., 2019). 1-heptadecanoyl-sn-glycero-3-phosphocholine, a lysophospholipid (LyP), originating from hydrolysis of phosphatidylcholine, can play an important role in the de-acylation/re-acylation cycle and then control molecular species composition (Lingwood and Simons, 2010). Watson identified that lipid metabolism played a physiological importance on atherosclerosis, diabetes, obesity, and Alzheimer’s disease (Watson, 2006). In our study, the level of acetylcarnitine was increased and 1-heptadecanoyl-sn-glycero-3-phosphocholine was decreased in VPs vs. SPs patients, which indicated that the two metabolites were involved in ACS. The more specific role of acetylcarnitine and 1-heptadecanoyl-sn-glycero-3-phosphocholine on the vulnerability of plaque stability needs to be further explored. And future studies should explore this relationship more fully, since it may lead to a non-invasive marker that differentiates VPs from SPs.

In this study, there are also some limitations that should be acknowledged. As isoundecylic acid was not found in the human metabolome database (HMDB), we only detect the three metabolites including in targeted LC/MS, and four metabolites including betaine, 1-heptadecanoyl-sn-glycero-3-phosphocholine, acetylcarnitine, and isoundecylic acid in untargeted LC/MS. In addition, the metabolite-based model was only validated by one center, but not validated from populations of external validation. However, the positive results from the model suggested a possible generalization of the combined model of serum betaine and EF in differentiating VPs from SPs.

In summary, the present study uncovered four metabolites which can separate patients with ACS from healthy controls. The combined model of serum betaine and EF model was proposed for the better diagnosis of the vulnerability of plaque stability in a non-invasive way.

## Data Availability Statement

All datasets generated for this study are included in the article/Supplementary Material.

## Ethics Statement

The studies involving human participants were reviewed and approved by the Ethics Committee of Tungwah Hospital of Sun Yat-sen University. The patients/participants provided their written informed consent to participate in this study.

## Author Contributions

JZ, BC, and HiJ conceived and designed the experiments. HiJ, HoJ, and SW performed the experiments. WC, CL, HL, and WH analyzed the data. JZ, BC, and HiJ wrote the manuscript. JZ, BC, and WH revised the manuscript for scientific content. All authors contributed to the article and approved the submitted version.

## Conflict of Interest

The authors declare that the research was conducted in the absence of any commercial or financial relationships that could be construed as a potential conflict of interest.

## Footnotes

1 https://metlin.scripps.edu

2 http://www.hmdb.ca

3 http://www.genome.jp/kegg

## Supplementary Material

TABLE S1 The 3069 metabolites detected by untargeted LC–MS.
